# Identification of matrix-remodeling associated 5 as a possible molecular oncotarget of pancreatic cancer

**DOI:** 10.1038/s41419-023-05684-5

**Published:** 2023-02-24

**Authors:** Shi-qing Peng, Xiao-ren Zhu, Ming-zhi Zhao, Yi-fan Zhang, An-ran Wang, Min-bin Chen, Zhen-yu Ye

**Affiliations:** 1 Department of Radiotherapy and Oncology, Affiliated Kunshan Hospital of Jiangsu University, Kunshan, China; 2 Clinical Research Center of Neurological Disease and Department of Orthopaedics, The Second Affiliated Hospital of Soochow University, Suzhou, China; 3 Department of General Surgery, The Second Affiliated Hospital of Soochow University, Suzhou, China

**Keywords:** Pancreatic cancer, Targeted therapies

## Abstract

Pancreatic cancer has an extremely poor prognosis. Here we examined expression, potential functions and underlying mechanisms of MXRA5 (matrix remodeling associated 5) in pancreatic cancer. Bioinformatics studies revealed that *MXRA5* transcripts are significantly elevated in pancreatic cancer tissues, correlating with the poor overall survival, high T-stage, N1 and pathologic stage of the patients. *MXRA5* mRNA and protein expression is significantly elevated in microarray pancreatic cancer tissues and different pancreatic cancer cells. In primary and immortalized (BxPC-3 and PANC-1 lines) pancreatic cancer cells, shRNA-induced MXRA5 silencing or CRISPR/Cas9-mediated MXRA5 knockout suppressed cell survival, proliferation, migration, invasion, and epithelial-mesenchymal transition (EMT), while provoking cell apoptosis. Conversely, forced overexpression of MXRA5 further promoted pancreatic cancer cell progression and EMT. Bioinformatics studies and the protein chip analyses revealed that differentially expressed genes (DEGs) and differentially expressed proteins (DEPs) in MXRA5-overexpressed primary pancreatic cancer cells were enriched in the PI3K-Akt-mTOR cascade. Indeed, Akt-mTOR activation in primary human pancreatic cancer cells was inhibited by MXRA5 shRNA or knockout, but was augmented following MXRA5 overexpression. In vivo, the growth of MXRA5 KO PANC-1 xenografts was largely inhibited in nude mice. Moreover, intratumoral injection of adeno-associated virus-packed MXRA5 shRNA potently inhibited primary pancreatic cancer cell growth in nude mice. Akt-mTOR activation was also largely inhibited in the MXRA5-depleted pancreatic cancer xenografts. Contrarily MXRA5 overexpression promoted primary pancreatic cancer cell growth in nude mice. Together, overexpressed MXRA5 is important for pancreatic cancer cell growth possibly through promoting EMT and Akt-mTOR activation. MXRA5 could be a potential therapeutic oncotarget for pancreatic cancer.

## Introduction

Pancreatic cancer is one of the most lethal malignancy worldwide [1, 2]. The 5-year overall survival of pancreatic cancer patients is less than 10% [3, 4]. Patients with advanced and/or metastatic pancreatic cancer often have extremely poor prognosis [1, 5]. Current treatment strategies, including surgery, radiotherapy and gemcitabine-based chemotherapy, failed to significantly improve survival and prognosis of advanced patients [6, 7]. It is often diagnosed at the advanced stages with cancer local infiltration and/or systematic metastasis [3]. Therefore, novel and more effective molecular-targeted therapies are urgently needed [8–10].

Extracellular matrix (ECM) is vital for cancer cell growth, metastasis and epithelial-to-mesenchymal cell transition (EMT) [11–14]. Cancer cells undergo invasion and metastasis through EMT that latter could be activated by ECM-related proteins and enzymes [15–17]. ECM also serves as nutrition source, promoting pancreatic cancer cell survival and proliferation under nutrition-low conditions [18]. Studies have shown that ECM is a key factor for tumorigenesis and the progression of pancreatic cancer [6, 7, 19].

Matrix-remodeling associated (MXRA) protein family proteins, including MXRA5, MXRA7 and MXRA8, are important in cell adhesion and ECM remodeling [20]. MXRA7 functions as a negative modulator in psoriasis development [21]. MXRA8 is a receptor for arthritogenic alphavirus [22]. MXRA5 is reported as a secreted glycoprotein, and it containes seven leucine-rich repeats and 12 immunoglobulin-like C2-type domains related to perlecan (Adlican) [23]. In osteoarthritis, MXRA5 was detected in the cartilage and synovial fluid, serving as an important protein for chondrocyte integrity and regeneration [24]. In the kidney, MXRA5 inhibited TGFβ-induced anti-inflammatory and anti-fibrotic activities by suppressing the expression of different chemokines, fibronectin and collagen [23, 25]. MXRA5 was identified as a novel ECM protein in calcified valves, and it participated in the initiation and progression of aortic stenosis [26]. Studies have reported MXRA5 upregulation in human malignancies, including non-small cell lung cancer (NSCLC), colorectal cancer (CRC), and glioma [27–29]. These studies have proposed a potential pro-cancerous activity of MXRA5 [27–29]. Here, we tested the expression, potential functions and underlying molecular mechanisms of MXRA5 in pancreatic cancer.

## Material and Methods

### Bioinformatics analysis

Oncomine (www.oncomine.org), the public database, was consulted to analyze *MXRA5* expression in various tumors including pancreatic adenocarcinoma (PAAD) and corresponding normal tissues [30]. *P*-value < 0.05 and fold change ≥ 1.5 was considered significant. GEPIA (http://gepia.cancer-pku.cn/) was searched for detecting *MXRA5* expression in pancreatic cancer tissues and normal pancreatic tissues [31, 32]. *MXRA5* transcripts from TCGA database (including 171 normal pancreatic tissues and 179 PAAD tissues) were analyzed and *MXRA5*-associated differentially expressed genes (DEGs) were retrieved. R software was used to do hierarchical clustering analysis and KEGG pathway analysis.

### Reagents

Fluorescence probes, including TUNEL (terminal deoxynucleotidyl transferase dUTP nick end labeling), tetraethylbenzimidazolylcarbocyanine iodide (JC*-*1), DAPI (4’,6-diamidino-2-phenylindole), LY294002, EdU (5-ethynyl-20-deoxyuridine), were provided by Thermo-Fisher Invitrogen (Shanghai, China). Polybrene, antibiotics, puromycin and medium were purchased from Sigma-Aldrich (St. Louis, MO). PF-562271 was provided by MCE China (Shanghai, China). The primary antibodies used were following: anti-MXRA5 (1:1000; LS‑C373823, LSBio, Shanghai, China), anti-MXRA7 (1:1000; TA336144, OriGene, Beijing, China), anti-β-actin (1:2000; Abcam), anti-GAPDH (1:2000; Cell Signaling Technology), anti-cleaved-poly (ADP-ribose) polymerase (PARP) E51 (1:1000; #32064; Abcam), anti-cleaved-Caspase 9 (1:1000; #2324; Abcam), anti-E-Cadherin (1:1000,#3195; Cell Signaling Technology), anti-N-Cadherin (1:1000; #245117;Abcam), anti-Vimentin (1:1000; #92547; Abcam), anti-p-Akt Ser-473 (1:1000; #4060; Cell Signaling Technology), anti-S6 (1:1000; #2217, Cell Signaling Technology), anti-phospho-S6 (1:1000; #4858, Cell Signaling Technology). Other antibodies were described previously [33–35].

### Cell culture and tissue microarray

The established pancreatic cancer cell lines, BxPC-3 and PANC-1, were purchased from Cell Bank of Shanghai Institute of Biological Science (Shanghai, China). Cells were cultivated under DMEM/F-12/RPMI plus 10% FBS. The detailed protocols of obtaining and culturing of primary human pancreatic cancer cells (“priPC-1”) and primary human pancreatic epithelial cells (“pEpi”) were reported previously [33]. Cells were routinely subject to mycoplasma and microbial contamination examination. The human pancreatic cancer tissue microarray was carried out by Shanghai SuperChip Co (Shanghai, China). The protocols of using human cells were approved by the Ethics Committee of Jiangsu University, in accordance to the Declaration of Helsinki.

### Cellular functional studies and gene/protein detection

Cell counting kit-8 (CCK-8) viability, colony formation, nuclear EdU /DAPI double staining (testing cell proliferation); “Transwell” cell migration and “Matrigel Transwell” cell invasion, as well as phagokinetic track motility assay, propidium iodide (PI)-FACS (testing cell cycle progression), the caspase-3/the caspase-9 activity, Annexin V-PI flow cytometry, TUNEL/DAPI double staining and JC-1 staining of mitochondrial depolarization were described in detail in our previous studies [34, 36, 37]. The detailed protocols of Western blotting and quantitative real-time PCR (qRT-PCR) were also reported early [34, 37, 38]. For the functional studies, cells with the applied genetic modifications or treatments were first cultivated for indicated time periods. Then, different reagents, including CCK-8, caspase substrates and Annexin V-PI, were added for 1 h [34]. Alternatively, fluorescence dyes, including EdU (2 h), JC-1 (20 min), and TUNEL (1 h), were added. Thereafter different functional assays were performed. The uncropped blotting images were presented in Figure S4.

### MXRA5 shRNA or overexpression

The MXRA5 shRNAs (“MXRA5-sh-S1/S2”, containing two different verified sequences, Genechem) or the MXRA5 cDNA was sub-cloned into a GV248 construct (no GFP, from Genechem). The construct, alongside with the lentivirus package constructs (also provided by Genechem), were transfected to HEK-293T virus packaging cells, and thereby generating MXRA5 shRNA lentiviral particles or MXRA5-overexpression lentiviral particles. The viral particles were then filtered, enriched and added (at MOI = 10) to cultured cells (seeded into six-well culture plates at 60–70% of confluence). Through puromycin selection (for 72 h), stable cells were selected and MXRA5 silencing or overexpression was verified by qRT-PCR and Western blotting assays. The scramble control shRNA lentiviral particles (“c-sh”) or the empty vector (“Vec”) lentiviral particles were added to control cells. For the in vivo xenograft studies, MXRA5-sh-S1 or the scramble control shRNA was cloned into an adeno-associated virus (aav) construct (aav9, Genechem). The shRNA virus was then generated.

### MXRA5 knockout (KO)

The pancreatic cancer cells were first transfected with the pLV-hUbC-dCas9-VP64 lentiviral construct (GeneChem), and single stable dCas9-expressing cells were established after selection using the puromycin-containing medium [39]. Next, the small guide RNA (sgRNA)-CRISPR/dCas-9 MXRA5-KO lentiviral construct, provided by Genechem, was transduced to dCas9-expressing pancreatic cancer cells, with stable cells established by using puromycin-containing medium for additional 96 h. MXRA5 KO was verified in the stable cells. The control pancreatic cancer cells were transfected with a lenti-CRISPR/dCas-9 empty vector with non-sense sgRNA (“Cas9-C”).

### Xenograft model

Animal protocols have been approved by Institutional Animal Care and Use Committee (IACUC) and the Ethics Review Board of The Affiliated Kunshan Hospital of Jiangsu University. Five- to six-week-old female nude mice were purchased from the Animal Center of Jiangsu University and were raised indoors at standard conditions. The described PANC-1 cells or the primary human pancreatic cancer cells (at three/six million cells per mouse) were subcutaneously injected into the flanks (in the upper limbs) of the nude mice. The mice body weights and tumor volumes were measured with digital calipers. Immunohistochemistry (IHC) protocols were reported previously [33].

### Statistical analysis

In vitro experiments were repeated five times. Data were always with normal distribution and were presented as mean ± standard deviation (SD). Statistical analysis was performed using SPSS 23.0 (SPSS Co., Chicago, IL). The significance between two treatment groups was tested by two-tailed student’s t-test. One-way ANOVA with the Scheffe’ and Tukey Test was employed for comparison of more than two groups. *P* values <0.05 were considered statistically significant.

## Results

### MXRA5 is overexpressed in human pancreatic cancer

First, the Oncomine database (www.oncomine.org) was consulted, and *MXRA5* expression in pancreatic cancer tissues was analyzed from the Badea pancreas dataset. As shown, the number of *MXRA5* transcripts in pancreatic cancer tissues is significantly higher than that in the normal pancreatic tissues (Figure S1A, Fold change = 5.430, *P* = 2.17E-15). In addition, the GEPIA online tool (https://www.gepia.cancer-pku.cn) was consulted to examine *MXRA5* expression. Results again show that *MXRA5* expression is remarkably elevated in pancreatic cancer (PAAD) tissues (Figure S1B, blue box), while its expression is relative low in normal pancreatic tissues (Figure S1B).

**Fig. 1:**
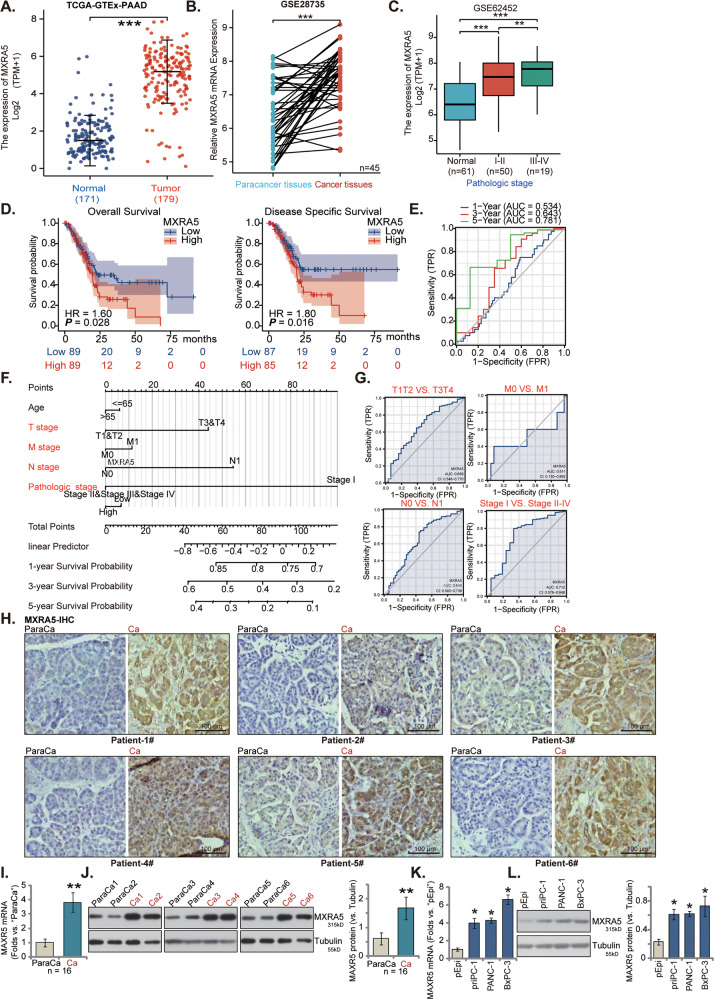
MXRA5 is overexpressed in pancreatic cancer. TCGA-GTEx-PAAD cohorts show the relative *MXRA5* mRNA transcripts in 179 cases of pancreatic ductal adenocarcinoma (PDAC) tissues (“Tumor”) and 171 cases of normal pancreatic tissues (“Normal”) (**A**); GEO dataset (GSE28735) showed that the number of MRXA5 transcripts in 45 PDAC tissues and matched adjacent paracancer tissues (**B**). GEO dataset (GSE62452) showed the number of MRXA5 transcripts in the normal pancreatic tissues (“Normal”) and in pancreatic cancer tissues with different clinical-pathological stage (**C**). The Kaplan Meier Survival curve of *MXRA5*-low (blue) and *MXRA5*-high (red) pancreatic cancer patients was presented (**D**). Subgroup analyses, based on the different clinical features of the pancreatic cancer patients, were performed as well (**E–G**). The representative MXRA5 IHC images of six PDAC patients (“Patient 1#” and “Patient 6#”) were presented (**H**). *MXRA5* mRNA and protein expression in pancreatic cancer tissues (“Ca”) and THE matched paracancer tissues (“ParaCa”) of sixteen (*n* = 16) different PDAC primary patients was shown (**I** and **J**). *MXRA5* mRNA and protein expression in the listed pancreatic cancer cells and primary human pancreatic ductal epithelial cells (“pEpi”) was tested (**K** and **L**). “TPM” stands for transcripts per million. “TRP” stands for “true positive rate”. “FRP” stands for “false positive rate” *P < 0.05, **P < 0.01, ***P < 0.01. Scale bar = 100 μm.

Next, the gene expression profile of 171 normal pancreatic tissues and 179 pancreatic cancer tissues were retrieved from TCGA-PAAD and GTEx databases. The number of *MXRA5* transcripts in pancreatic cancer tissues is significantly higher than that in the normal pancreatic tissues (Fig. 1A, *P* < 0.001). GEO dataset (GSE28735) analyses of TCGA data showed that the number of MRXA5 transcripts in 45 different pancreatic ductal adenocarcinoma (PDAC) tissues was significantly higher than that in the matched adjacent paracancer tissues (Fig. 1B, *P* < 0.001). In addition, GEO dataset (GSE62452) showed that MXRA5 overexpression is positively correlated with higher tumor clinical pathological stage (Stage-I&II vs. Stage III & IV; *P* < 0.01) (Fig. 1C).

Kaplan-Meier survival plus univariate Cox analysis confirmed that high *MXRA5* expression in pancreatic cancer was correlated with the poor prognosis of the patients (Fig. 1D). Moreover, *MXRA5* overexpression could be a risk factor for poor overall survival [hazard ratio (HR): 1.60, *P* = 0.028] (Fig. 1D) and disease-specific survival [DSS, hazard ratio (HR): 1.80, *P* = 0.016] (Fig. 1D). The receiver operating characteristic (ROC) curve results imply that *MXRA5* overexpression could have a potential predictive value on the 1-/3-/5-year survival of pancreatic cancer patients. Area Under Curve (AUC) values were 0.534, 0.643 and 0.781 for 1-year, 3-year and 5-year survival predictions (Fig. 1E, F). ROC curves were applied to verify the accuracy of the model according to *MXRA5* expression in pancreatic cancer tissues and clinicopathological characteristics of the patients (Fig. 1F, G). *MXRA5* overexpression in pancreatic cancer was correlated with higher T classification (AUC = 0.659), N classification (AUC = 0.640) and clinical pathological stage (AUC = 0.712) (Fig. 1F, G). These bioinformatics studies confirmed elevated *MXRA5* expression in human pancreatic cancer.

The representative immunohistochemistry (IHC) images of six patients (“Patient 1#” and “Patient 6#”) demonstrated that MXRA5 protein expression in the pancreatic cancer tissues (“Ca”) was higher than that in the paracancer pancreatic tissues (“ParaCa”) (Fig. 1H). Moreover, the fresh pancreatic cancer tissues and matched paracancer tissue of a total of sixteen (n = 16) different primary patients were obtained at the time surgery. As shown, *MXRA5* mRNA expression in pancreatic cancer tissues (“Ca”) was again significantly higher than that in paracancer tissues (“ParaCa”) (Fig. 1I). MXRA5 protein upregulation was also detected in pancreatic cancer tissues of six representative patients (“Patient 1#” and “Patient 6#”) (Fig. 1J). After combining all 16 pairs of blotting data, results showed that MXRA5 protein upregulation in pancreatic cancer tissues was significant (*P* < 0.05 vs. “PareCa” tissues) (Fig. 1J).

The expression of MXRA5 in human pancreatic cancer cells was studied. The primary pancreatic cancer cells (“priPC-1”) [34], the established pancreatic cancer cell lines (PANC-1 and BxPC-3) and the normal primary human pancreatic epithelial cells (“pEpi”) [34] were cultured and tested. As shown, *MXRA5* mRNA (Fig. 1K) and protein (Fig. 1L) expression in different pancreatic cancer cells was significantly elevated when compared to its expression in pEpi cells. These results clearly showed that MXRA5 is overexpressed in human pancreatic cancer.

### MXRA5 shRNA induces robust antipancreatic cancer cell activity

**Fig. 2:**
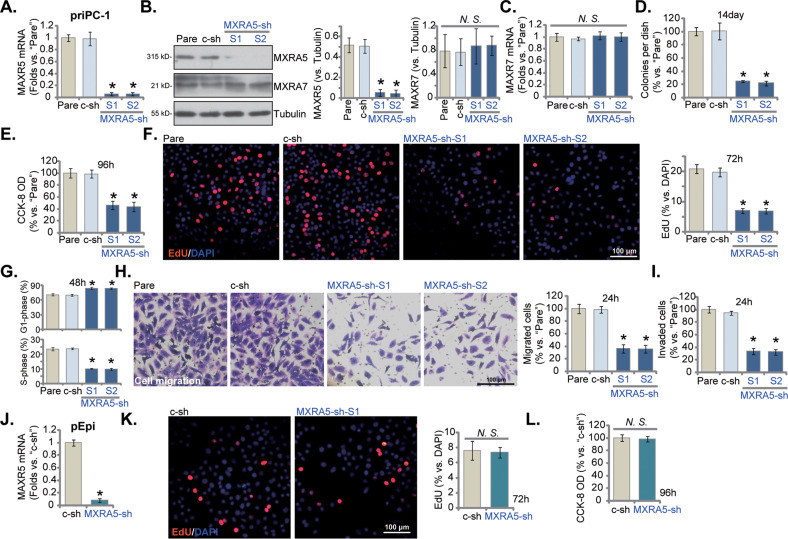
MXRA5 shRNA induces robust anti-pancreatic cancer cell activity. Puromycin-selected priPC-1 primary human pancreatic cancer cells, with the applied MXRA5 shRNA (“MXRA5-sh-S1/S2”, containing two different sequences) or the scramble control shRNA (“c-sh”), were cultured, and expression of listed genes and proteins was shown (**A**–**C**); Cells were further cultivated for indicated time periods, colony formation (**D**), cell viability (**E**) and EdU incorporation (**F**) as well as cell cycle progression (**G**), in vitro cell migration (**H**) and invasion (**I**) were tested using the described methods. Puromycin-selected pEpi primary human pancreatic epithelial cells, expressing MXRA5-sh-S1 (“MXRA5-sh”) or “c-sh”, were established. *MXRA5* mRNA expression was shown (**J**). Cells were further cultivated for indicated time periods, cell proliferation (nuclear EdU staining, **K**) and CCK-8 viability (**L**) were examined. “Pare” stands for the parental control cells. Error bars stand for mean ± standard deviation (SD, *n* = 5). *P < 0.05 versus “Pare”/“c-sh” cells. “*N.S*.” stands for *P* > 0.05. Experiments in this figure were repeated five times. Scale bar = 100 μm.

To explore the potential function of MXRA5 in pancreatic cancer cells, the lentiviral particles encoding MXRA5 shRNA (“MXRA5-sh-S1/S2”, containing different targeting sequences) were added to primary human pancreatic cancer cells (“priPC-1”). After selection by puromycin the stable cells were formed. Control cells were stably transduced with scramble control shRNA lentiviral particles (“c-sh”). *MXRA5* mRNA levels decreased over 90% in stable priPC-1 cells with MXRA5-sh-S1/S2 (Fig. 2A). MXRA5 protein downregulation was also detected in MXRA5-sh-expressing priPC-1 cells (Fig. 2B), where *MXRA7* mRNA and protein expression was unchanged (Fig. 2B, C). shRNA-induced silencing of MXRA5 decreased the number of viable priPC-1 cell colonies (Fig. 2D) and inhibited CCK-8 cell viability (Fig. 2E). The applied MXRA5 shRNAs inhibited priPC-1 cell proliferation and decreased EdU-positive nuclei ratio in priPC-1 cells (Fig. 2F). Moreover, MXRA5 silencing disrupted priPC-1 cell cycle progression by increasing G1-phase percentage while decreasing S-phase percentage, leading to G1-S arrest (Fig. 2G). Next, in vitro cell migration and invasion were examined through “Transwell” and “Matrigel Transwell” assays, respectively. MXRA5 shRNA slowed priPC-1 cell in vitro migration (Fig. 2H) and invasion (Fig. 2I).

Similarly in the immortalized PANC-1 cells and BxPC-3 cells, MXRA5 *mRNA* (Figure S2A) and protein (Figure S2B) expression was significantly decreased after treatment with the MXRA5-sh-S1 lentiviral particles (“MXRA5-sh”). MXRA5-sh failed to alter MXRA7 protein expression (Figure S2B). The numbers of PANC-1 cell colonies and BxPC-3 cell colonies were significantly decreased after MXRA5 silencing (Figure S2C). MXRA5 silencing also hindered PANC-1 and BxPC-3 cell proliferation and the EdU-positive nuclei ratio was significantly decreased (Figure S2D). In addition, MXRA5 shRNA slowed PANC-1 cell in vitro migration (Figure S2E) and invasion (Figure S2F). The phagokinetic track motility assay results demonstrated that MXRA5 silencing inhibited the motility of PANC-1 cells (Figure S2G) and BxPC-3 cells (Figure S2H). In the primary human pancreatic epithelial cells (“pEpi”), the lentiviral MXRA5-sh-S1 (“MXRA5-sh”) similarly resulted in robust *MXRA5* mRNA silencing (Fig. 2J). However, it failed to affect pEpi cell proliferation (EdU incorporation, Fig. 2K) and CCK-8 viability (Fig. 2L), supporting a cancer cell-specific effect by MXRA5 silencing. Collectively, MXRA5 shRNA resulted in robust anti-pancreatic cancer cell activity, inhibiting cell viability, proliferation, cell cycle progression and motility.

### MXRA5 knockout potently inhibits pancreatic cancer cell progression in vitro

Next, the sgRNA-CRISPR/dCas-9 MXRA5-KO lentiviral construct was transduced to dCas9-expressing priPC-1 cells. After puromycin-mediated selection and MXRA5 KO verification, the single stable priPC-1 cells (“MXRA5-ko”) were established, showing depleted *MXRA5* mRNA and protein expression (Figure S3A, B). *MXRA7* mRNA and protein expression was unchanged (Figure S3B, C). MXRA5 KO inhibited CCK-8 viability (Figure S3D) in priPC-1 primary cells. In addition, MXRA5 KO robustly inhibited priPC-1 cell proliferation and decreased EdU-positive nuclei ratio in priPC-1 cells (Figure S3E). Evidenced from results of “Transwell” and “Matrigel Transwell” assays, MXRA5 KO robustly hindered priPC-1 in vitro cell migration (Figure S3F) and invasion (Figure S3G). In the primary human pancreatic epithelial cells (“pEpi”), CRISPR/Cas9 strategy was also utilized to induce *MXRA5* mRNA depletion (Figure S3H). Yet it failed to alter cell viability (CCK-8 OD, Figure S3I) and proliferation (EdU incorporation, Figure S3J). Together, these results revealed that CRISPR/Cas9-induced MXRA5 KO induced potent anti-pancreatic cancer cell activity.

### MXRA5 depletion provokes apoptosis in pancreatic cancer cells

**Fig. 3:**
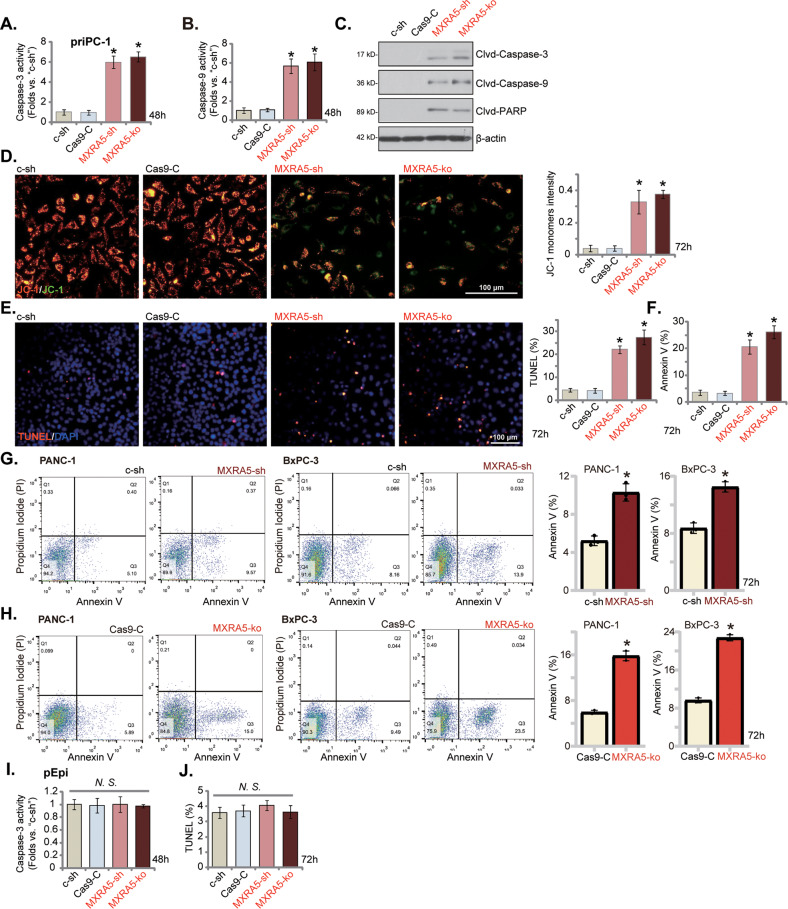
MXRA5 silencing provokes apoptosis in pancreatic cancer cells. Puromycin-selected priPC-1 primary human pancreatic cancer cells (**A**-**F**) or the established cell lines (PANC-1 and BxPC-3, **G** and **H**) or pEpi primary human pancreatic epithelial cells (**I** and **J**), with the MXRA5-sh-S1 (“MXRA5-sh”), the scramble control shRNA (“c-sh”), the sgRNA-CRISPR/dCas-9 MXRA5-KO lentiviral construct (“MXRA5-ko”) or Cas9 control construct (“Cas9-C”), were cultured for applied time periods, and the caspase-3 activity and the caspase-9 activity were tested (**A**, **B** and **I**); Expression of listed proteins was shown (**C**). Mitochondrial depolarization was examined by JC-1 staining (**D**). Cell apoptosis was examined by nuclear TUNEL staining (**E** and **J**) and Annexin V flow cytometry (**F**–**H**) assays. Error bars stand for mean ± standard deviation (SD, *n* = 5). *P < 0.05 versus “c-sh” cells. “Clvd” stands for “cleaved”. “*N.S*.” stands for *P* > 0.05. Experiments in this figure were repeated five times. Scale bar = 100 μm.

MXRA5 shRNA/KO inhibited pancreatic cancer proliferation, cell cycle progression, and mobility, we next tested its potential activity on cell apoptosis. First we examined the caspase activities in MXRA5-depleted cells. In priPC-1 cells with MXRA5-sh-S1 (“MXRA5-sh”) or with MXRA5-ko (see Figure S3), the caspase-3 activity (Fig. 3A) and the caspase-9 activity (Fig. 3B) were robustly increased. Cleavages of caspase-3, caspase-9 and PARP1 were significantly increased in MXRA5-depleted priPC-1 cells (Fig. 3C). MXRA5 silencing or KO also resulted in mitochondrial depolarization in priPC-1 cells (Fig. 3D), causing JC-1 red fluorescence transition to green monomers (Fig. 3D). Further studies revealed that MXRA5 depletion induced apoptosis in priPC-1 cells and increased TUNEL-positive nuclei ratio (Fig. 3E). Further supporting apoptosis activation, we found that MXRA5 shRNA or KO increased Annexin V positively stained priPC-1 cell ratio (Fig. 3F). Similarly in PANC-1 and BxPC-3 cells, MXRA5 shRNA or KO robustly increased the number of Annexin V-positive staining (Fig. 3G, H). In the primary human pancreatic epithelial cells (“pEpi”), however, MXRA5 silencing or depletion failed to significantly increase the caspase-3 activity (Fig. 3I) and TUNEL-positive nuclei ratio (Fig. 3J). These results supported that MXRA5 depletion provoked apoptosis in pancreatic cancer cells.

### MXRA5 overexpression exerts pro-cancerous activity in pancreatic cancer cells

**Fig. 4:**
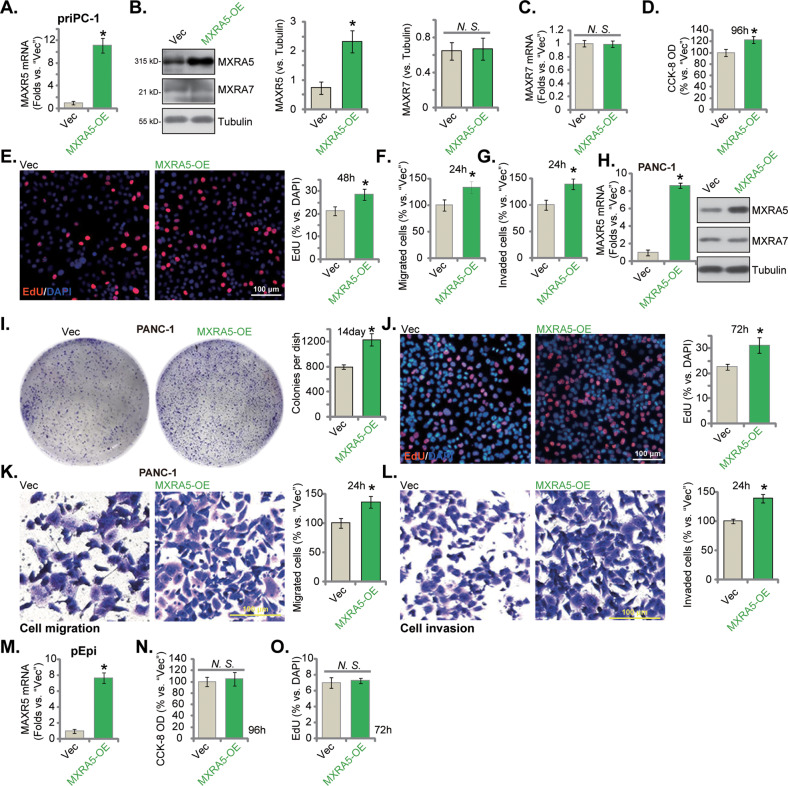
MXRA5 overexpression exerts pro-cancerous activity in pancreatic cancer cells. Puromycin-selected priPC-1 primary human pancreatic cancer cells (**A**–**G**), the established PANC-1 cells (**H**–**L**) or the pEpi primary human pancreatic epithelial cells (**M-O**) with the lentiviral MXRA5 overexpression construct (“MXRA5-OE”) or the corresponding vector (“Vec”) were formed and expression of listed genes and proteins was shown (**A**-**C**, **H** and **M**); Cells were further cultivated for indicated time periods, cell viability (**D** and **N**), colony formation (**I**) and EdU incorporation (**E**, **J** and **O**) as well as in vitro cell migration (**F** and **K**) and invasion (**G** and **L**) were tested using the described methods. Error bars stand for mean ± standard deviation (SD, *n* = 5). *P < 0.05 versus “Vec” cells. “*N.S*.” stands for *P* > 0.05. Experiments in this figure were repeated five times. Scale bar = 100 μm.

We next hypothesized that ectopic overexpression of MXRA5 could possibly exert pro-cancerous activity in pancreatic cancer cells. The lentiviral particles encoding *MXRA5* cDNA were added to priPC-1 primary cancer cells. After selection using puromycin-containing medium, MXRA5 overexpressed priPC-1 cells, “MXRA5-OE”, were formed. As compared to the vector control cells (“Vec”), *MXRA5* mRNA (Fig. 4A) and protein (Fig. 4B) levels were remarkably elevated in MXRA5-OE priPC-1 cells, and *MXRA7* mRNA (Fig. 4C) and protein (Fig. 4B) were unchanged. Overexpression of MXRA5 increased CCK-8 viability OD (Fig. 4D) and EdU incorporation (Fig. 4E) in priPC-1 cells. Moreover, priPC-1 cell in vitro migration (Fig. 4F) and invasion (Fig. 4G) were accelerated following ectopic MXRA5 overexpression.

Similarly in PANC-1 cells, ectopic overexpression of MXRA5 (“MXRA5-OE”) using the same lentiviral construct led to *MXRA5* mRNA and protein elevation (Fig. 4H). MXRA5-OE augmented colony formation (Fig. 4I) and EdU incorporation (Fig. 4J). Moreover, in vitro PANC-1 cell migration and invasion were accelerated after MXRA5 overexpression (Fig. 4K, L). Whereas in the primary human pancreatic epithelial cells (“pEpi”), ectopic MXRA5 overexpression by the same method (Fig. 4M) failed significantly increase cell viability (Fig. 4N) and proliferation (EdU incorporation, Fig. 4O), again supporting a cancer cell-specific effect by MXRA5.

### MXRA5 promotes epithelial-to-mesenchymal cell transition (EMT) in pancreatic cancer cells

**Fig. 5:**
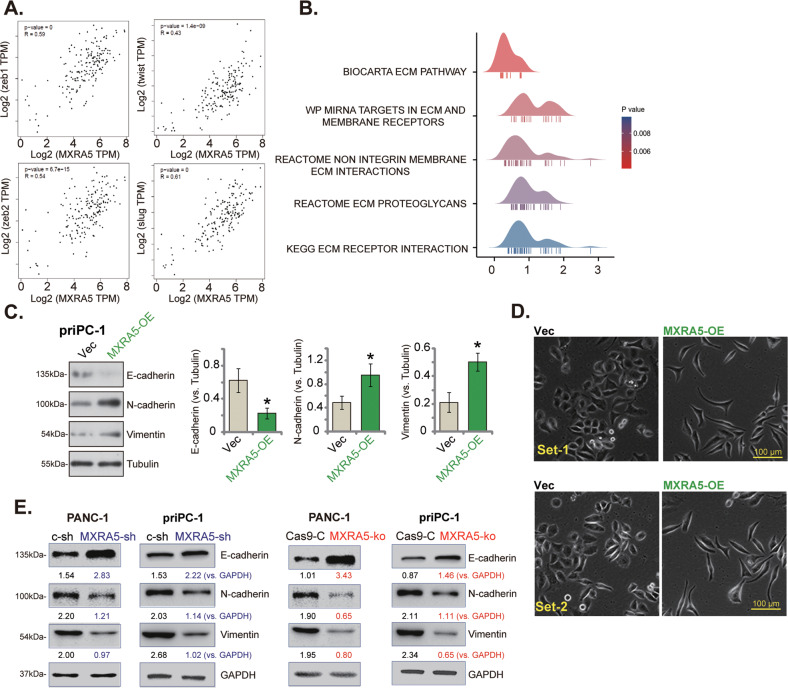
MXRA5 promotes epithelial-to-mesenchymal cell transition (EMT) in pancreatic cancer cells. Scatter plots of correlation between *MXRA5* mRNA expression and EMT marker genes from the TCGA-PAAD database (**A**). Ridge plot shows the correlation between *MXRA5* mRNA expression and ECM signature markers in pancreatic cancer (**B**). Puromycin-selected priPC-1 primary human pancreatic cancer cells or PANC-1 cells, with the lentiviral MXRA5 overexpression construct (“MXRA5-OE”), the corresponding vector (“Vec”), MXRA5-sh-S1 (“MXRA5-sh”), the scramble control shRNA (“c-sh”), the sgRNA-CRISPR/dCas-9 MXRA5-KO lentiviral construct (“MXRA5-ko”) or Cas9 control construct (“Cas9-C”), were cultured and expression of EMT marker proteins was tested (**C** and **E**). The characteristic EMT morphology in MARA5-overexpressed priPC-1 primary cancer cells were presented (**D**). Error bars stand for mean ± standard deviation (SD, *n* = 5). “TPM” stands for transcripts per million. “ECM” stands for extracellular matrix. *P < 0.05 versus “Vec” cells. Experiments in this figure were repeated five times. Scale bar = 100 μm.

MXRA5 is a key protein for cell adhesion and extracellular matrix (ECM) remodeling [26, 40, 41]. Our results revealed that MXRA5 was important for the migration and invasion of pancreatic cancer cells, it therefore could possibly influence EMT. EMT is executed by various EMT transcription factors (EMT-TFs) [14, 42, 43]. Based on *MXRA5* expression difference, we analyzed the expression of EMT-TFs under the GEPIA online tool (Fig. 5A). As shown, in pancreatic cancer tissues MXRA5 is co-expressed with a number of key ECM-TFs, including Zeb1, Zeb2, Twist and Slug [14, 42, 43]. Gene ontology analysis discovered that *MXRA5*-related DEGs were indeed enriched in a number of ECM process (Fig. 5B). Furthermore, E-Cadherin, the EMT marker protein, was downregulated in OE-MXRA5 priPC-1 cells, whereas N-Cadherin and vimentin were upregulated (Fig. 5C). Figure 5D demonstrated the characteristic EMT morphology in MARA5-overexpressed priPC-1 cells. Conversely, in both priPC-1 primary cancer cells and established PANC-1 cells, shRNA-mediated knockdown or CRISPR/Cas9-induced knockout of MXRA5 exerted opposite functions by upregulating E-Cadherin, but downregulating N-Cadherin and vimentin (Fig. 5E). These results implied that MXRA5 could be important for EMT in pancreatic cancer cells.

### MXRA5 is important for Akt-mTOR activation in pancreatic cancer cells

**Fig. 6:**
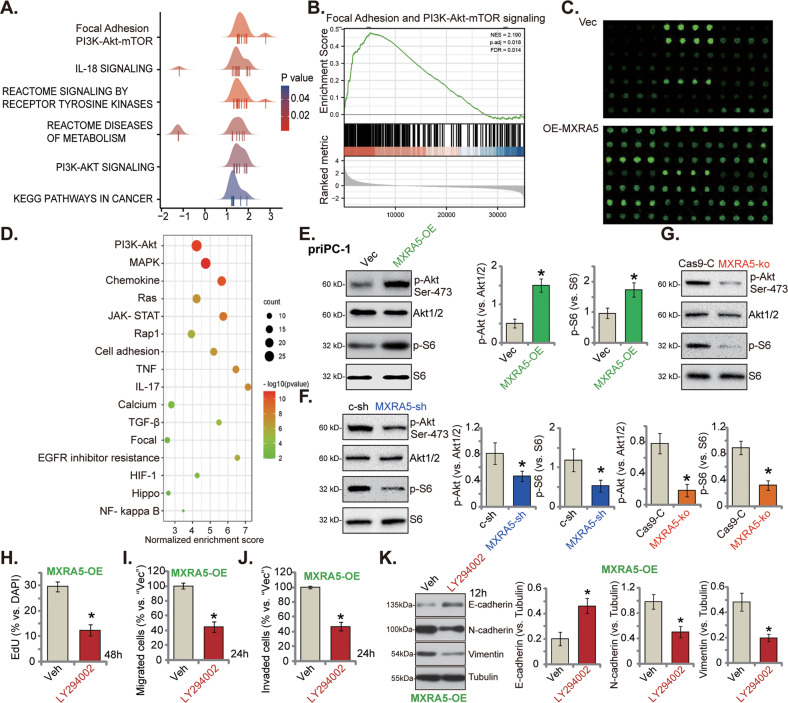
MXRA5 is important for PI3K-Akt-mTOR activation in pancreatic cancer cells. KEGG pathway plus Gene Set Enrichment Analysis (GSEA) analyses of MXRA5-associated differentially expressed genes (DEGs) and enriched pathways from the TCGA-PAAD database (**A** and **B**). Protein chip study of differentially expressed proteins (DEPs) in priPC-1 primary human pancreatic cancer cells with the lentiviral MXRA5 overexpression construct (“MXRA5-OE”) or the corresponding vector (“Vec”) (**C**); KEGG pathway analysis on the MXRA5-associated DEPs was shown (**D**). Puromycin-selected priPC-1 cells with MXRA5-OE, Vec, MXRA5-sh-S1 (“MXRA5-sh”), the scramble control shRNA (“c-sh”), the sgRNA-CRISPR/dCas-9 MXRA5-KO lentiviral construct (“MXRA5-ko”) or Cas9 control construct (“Cas9-C”), were cultured, and expression of listed proteins was shown (**E**-**G**). The OE-MXRA5 priPC-1 cells were treated with the PI3K-Akt-mTOR pan inhibitor LY294002 (5 μM) or the vehicle control (0.1% DMSO, “Veh”) for indicated time periods, EdU incorporation (**H**), in vitro cell migration (**I**) and invasion (**J**) were tested, with results quantified; Expression of listed proteins was shown (**K**). Error bars stand for mean ± standard deviation (SD, *n* = 5). *P < 0.05 versus “Vec”/“c-sh”/“Cas9-C” cells or “Veh” treatment. Experiments in this figure were repeated five times.

To further explore the possible mechanism of MXRA5-driven pancreatic cancer progression, KEGG pathway enrichment analysis and Gene Set Enrichment Analysis (GSEA) were employed to analyze differentially expressed genes (DEGs) and enriched pathways in MXRA5-overexpressed pancreatic cancer tissues from the TCGA-PAAD database. MXRA5-associated DEGs were enriched in the multiple pro-cancerous cascades, including focal adhesion, PI3K-Akt-mTOR signaling and IL-18 signaling pathway (Fig. 6A, B). Protein chip analysis was then carried out to analyze differentially expressed proteins (DEPs) between OE-MXRA5 priPC-1 primary cancer cells and vector control cells. DEPs of OE-MXRA5 cells were again enriched in PI3K-Akt-mTOR signaling pathway, JAK-STAT signaling pathway and cell adhesion molecules (Fig. 6C, D). These results implied that MXRA5 could be important for the activation of PI3K-Akt-mTOR cascade, a key signaling driving pancreatic cancer progression [9, 10, 44–47]. Indeed, ectopic overexpression of MXRA5 in priPC-1 primary cancer cells increased phosphorylation of Akt (Ser-473) and S6 (Fig. 6E). Conversely, shRNA-induced knockdown or CRISPR/Cas9-induced knockout of MXRA5 suppressed the phosphorylations of Akt (Ser-473) and S6 in priPC-1 primary cancer cells (Fig. 6F, G). Therefore MXRA5 is important for Akt-mTOR activation in pancreatic cancer cells.

Treatment with LY294002, the PI3K-Akt-mTOR pan inhibitor [48], largely inhibited proliferation (EdU incorporation, Fig. 6H), migration (Fig. 6I) and invasion (Fig. 6J) in OE-MXRA5 priPC-1 cells. Moreover, N-Cadherin and vimentin protein downregulation but E-Cadherin protein upregulation were detected in LY-294002-treated OE-MXRA5 priPC-1 cells (Fig. 6K). These results supported that Akt-mTOR activation is important for MXRA5-driven priPC-1 cell proliferation, mobility and EMT.

Considering that MXRA5-associated DEGs are enriched in focal adhesion (Fig. 6A, B) and activation of focal adhesion kinase (FAK) is important for Akt-mTOR activation and pancreatic cell growth [49, 50]. We tested whether MXRA5 is important for FAK activation in pancreatic cancer cells. Results showed that MXRA5 silencing by MXRA5-sh-S1 (“MXRA5-sh”) or MXRA5-ko potently inhibited FAK phosphorylation in priPC-1 cells (Figure S5A). Contrarily, in OE-MXRA5 priPC-1 cells phosphorylation of FAK was increased (Figure S5B). Importantly, treatment with the well-known FAK inhibitor PF-562271 [51, 52] largely inhibited Akt-S6 phosphorylation in OE-MXRA5 priPC-1 cells (Figure S5C). These results supported that MARA5-mediated Akt-mTOR activation could be through activating FAK.

### MXRA5 KO suppresses PANC-1 xenograft growth in vivo

**Fig. 7:**
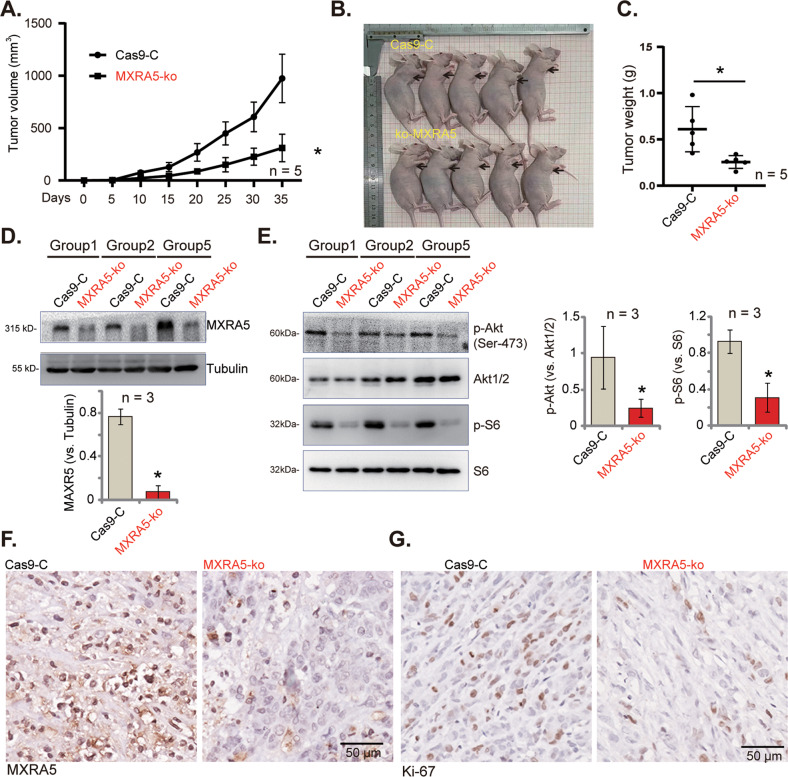
MXRA5 KO suppresses PANC-1 xenograft growth in vivo. PANC-1 xenografts were established by subcutaneous injection of PANC-1 cells (6 × 10^6^ cells per mouse), expressing the sgRNA-CRISPR/dCas-9 MXRA5-KO lentiviral construct (“MXRA5-ko”) or Cas9 control construct (“Cas9-C”), to the nude mice, and tumor volumes were recorded every five days (**A**). After 35 days, PANC-1 xenografts were measured (**B**) and weighted (**C**). The tumor tissue lysates were obtained, and expression of listed proteins was tested by Western blotting assays (**D** and **E**), with results quantified. The representative IHC images showed MXRA5 (**F**) and Ki-67 (**G**) expression in the xenograft tissue slides. Data were presented as mean ± standard deviation (SD). *P < 0.05 vs. “Cas9-C” tumors. Scale bar = 50 μm.

To examine MXRA5’s role on pancreatic cancer cell growth in vivo, ko-MXRA5 PANC-1 cells and the Cas9-C control cells were subcutaneously injected to the right flanks of the nude mice (at six million cells per mouse, five mice per group/n = 5). The tumor volumes were then recorded every five days, from “Day-0” to “Day-35”. The tumor growth curve results showed that the volumes of ko-MXRA5 PANC-1 xenografts were significantly lower than those of the Cas9-C control PANC-1 xenografts (Fig. 7A). At Day-35, all mice were euthanized and the xenografts were isolated and weighted. As shown, ko-MXRA5 PANC-1 xenografts were significantly smaller and lighter than Cas9-C control PANC-1 xenografts (Fig. 7B, C). The mice body weights were not significantly different between the two groups. We failed to notice any apparent toxicities in the experimental nude mice. The fresh tumor tissues were analyzed, we found that MXRA5 protein expression was significantly decreased in tissues of three ko-MXRA5 PANC1 xenografts (Fig. 7D), where p-Akt (Ser-473) and p-S6 were significantly inhibited (Fig. 7E). The representative IHC images further confirmed MXRA5 protein depletion in ko-MXRA5 xenograft slides (Fig. 7F). The decrease of Ki-67 staining in the representative MXRA5-ko PANC1 xenograft slide supported proliferation inhibition in vivo (Fig. 7G). Together, MXRA5 KO inhibited PANC-1 xenograft growth in vivo.

### MXRA5 silencing inhibits primary pancreatic cancer cell growth in vivo

**Fig. 8:**
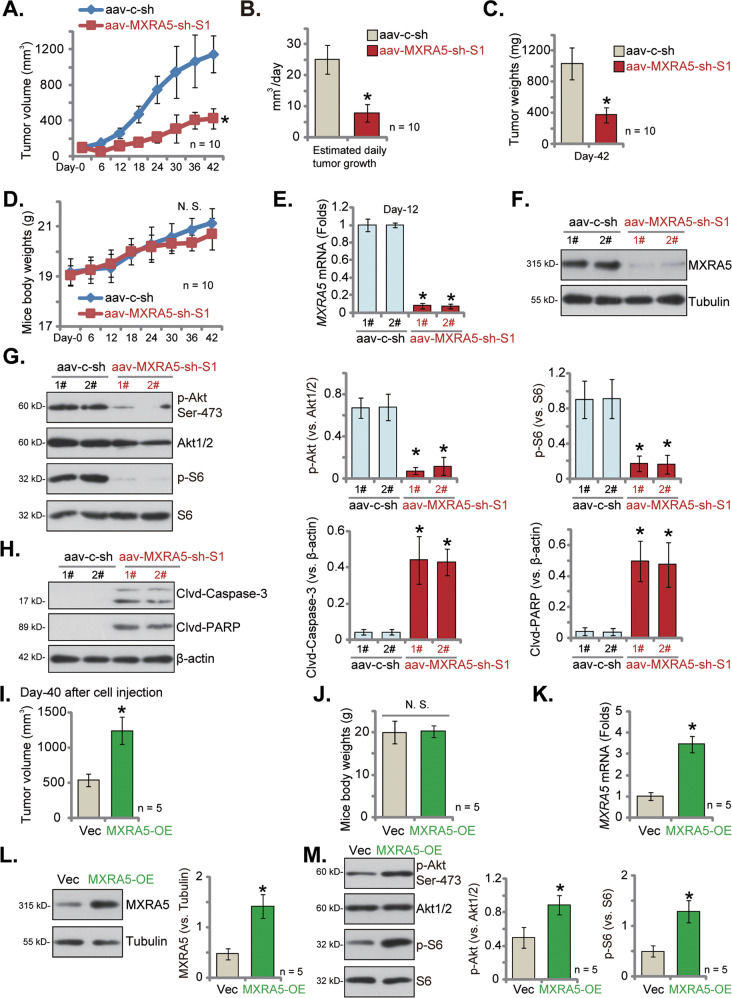
MXRA5 silencing inhibits primary pancreatic cancer cell growth in vivo. At 3 × 10^6^ cells per mouse, priPC-1 primary pancreatic cancer cells were *s.c*. injected to the nude mice and xenograft tumors (close to 100 mm^3^ in volume of each tumor) were formed after 20 days (labeled as “Day-0”). Mice were then subject to daily (from “Day-0” to “Day-9”) intratumoral injection of adeno-associated virus (aav)-packed MXRA5-sh-S1 (“aav-MXRA5-sh-S1”) or aav-packed scramble control shRNA (“aav-c-sh”); Tumor volumes (**A**) and mice body weights (**D**) were recorded every six days. The estimated daily tumor growth (in mm^3^ per day) was calculated (**B**); At Day-42, the priPC-1 xenografts were isolated and weighted (**C**). Expression of listed genes and proteins in the described priPC-1 xenograft tissues were tested (**E**-**H**). At 3 × 10^6^ cells per mouse, priPC-1 primary pancreatic cancer cells with the lentiviral MXRA5 overexpression construct (“MXRA5-OE”) or the corresponding vector (“Vec”) were *s.c*. injected to the nude mice, and xenograft tumors isolated after 40 days. Tumor volumes (**I**) and mice body weights (**J**) were recorded. Expression of listed genes and proteins in the described priPC-1 xenograft tissues were tested (**K**–**M**). Data were presented as mean ± standard deviation (SD). *P < 0.05 vs. “aav-c-sh”/“Vec” group. “*N.S*.” stands for *P* > 0.05.

At last, we tested whether MXRA5 silencing could inhibit primary pancreatic cancer cell growth in vivo. Therefore, priPC-1 primary cancer cells, at three million cells per mouse, were *s.c*. injected to the nude mice. After 20 days, the priPC-1 xenografts were formed and the volume of each tumor was close to 100 mm^3^ (it was labeled as “Day-0”). The xenograft-bearing mice were then subject to intratumoral injection of adeno-associated virus (aav)-packed MXRA5-sh-S1 (“aav-MXRA5-sh-S1”) or aav-packed scramble control shRNA (“aav-c-sh”). AAV was injected daily for 10 consecutive days. Figure 8A demonstrated that aav-MXRA5-sh-S1 injection robustly suppressed priPC-1 xenograft growth in nude mice. The volumes of aav-MXRA5-sh-S1 priPC-1 xenografts were significantly lower than the aav-c-sh control xenografts (Fig. 8A). The estimated daily tumor growth, in mm^3^ per day, was calculated (using the described formula [53]) and results showed that aav-MXRA5-sh-S1 injection robustly inhibited priPC-1 xenograft growth in mice (Fig. 8B). At experimental Day-42, all mice were decapitated and priPC-1 xenografts were carefully isolated and weighted. The aav-MXRA5-sh-S1-injected priPC-1 xenografts were remarkably lighter than the aav-c-sh-injected xenografts (Fig. 8C). We failed to detect significant difference in the mice body weights among the two groups (Fig. 8D). Therefore, intratumoral injection of MXRA5 shRNA virus remarkably hindered primary pancreatic cancer xenograft growth in vivo.

Signalings in the xenograft tissues were examined as well. At Day-12, two mice (“1#/2#”) per group were decapitated and priPC-1 xenograft tissues were surgically separated. Each tumor was cut into five small random pieces and tumor tissue lysates were tested. As shown, *MXRA5* mRNA (Fig. 8E) and protein (Fig. 8F) expression were dramatically decreased in aav-MXRA5-sh-S1-injected xenograft tissues, where p-Akt (Ser-473) and p-S6 were both dramatically inhibited (Fig. 8G). Conversely, cleavages of caspase-3 and PARP were significantly increased in aav-MXRA5-sh-S1 priPC-1 xenograft tissues (Fig. 8H). These results implied that MXRA5 shRNA virus injection silenced MXRA5, inhibited Akt-mTOR activation and provoked apoptosis in priPC-1 xenografts.

### MXRA5 overexpression promotes primary pancreatic cancer cell growth in vivo

Lastly, we assessed of the effect of MXRA5 overexpression in pancreatic cell growth in vivo. The priPC-1 cells with the lentiviral MXRA5 overexpression construct (“MXRA5-OE”) or the corresponding vector (“Vec”) were *s.c*. injected to the flanks of the nude mice. After 40 days, priPC-1xenografts were isolated and measured (Fig. 8I). As shown, the volumes of MXRA5-OE priPC-1 xenografts were significantly higher than those of Vec priPC-1 xenografts (Fig. 8I). No significant difference in the mice body weights was detected (Fig. 8J). *MXRA5* mRNA (Fig. 8K) and protein (Fig. 8L) expression was significantly increased in MXRA5-OE priPC-1 xenograft tissues. Moreover, phosphorylation of Akt (Ser-473) and S6 was increased as well (Fig. 8M). These results further support that MXRA5 overexpression promotes pancreatic cancer cell growth in vivo.

## Discussion

The potential function of MXRA5 in human cancer and its underlying mechanisms are largely unknown [27–29]. MXRA5 is a secreted glycoprotein regulating cell adhesion and extracellular matrix remodeling [40]. MXRA5 is expressed in primates and mammals, but not in mice [40]. The subcutaneous fibroblasts cDNA array results revealed that *MXRA5* was upregulated in subcutaneous fibroblasts after ionizing radiation, which could be associated with radiation-induced fibrosis (RIF) [54, 55]. *MXRA5* expression is elevated in skin fibroblasts from centenarians compared with that in younger controls [56].

MXRA5 could be important for the tumorigenesis and progression of human cancer, including non-small cell lung cancer (NSCLC), colorectal cancer, breast cancer and glioma. Xiong et al., have shown that somatic mutations of MXRA5 are observed in patients with NSCLC, which is possibly involved in the altered ECM remodeling and the etiology of NSCLC [27]. Wang et al., reported that MXRA5 is aberrantly expressed in colorectal cancer (CRC) tissues and could be a potential biomarker for the early diagnosis and prognosis of CRC [28].

Minafra et al., revealed that MXRA5 is upregulated in breast cancer, important for the EMT progression and matrix remodeling [57]. Sun et al., found that MXRA5 overexpression is correlated with multiple clinicopathologic features of human glioma, including histological grade, immune checkpoint molecule expression and tumor-associated macrophage infiltration [29]. Buckanovich et al. discovered that *MXRA5* mRNA levels were elevated in ovarian cancer, and it was possibly associated with tumor angiogenesis [58]. However, these studies failed to explored the inside molecular mechanisms of MXRA5 in tumorigenesis. Moreover, MXRA5’s expression and biological functions in human pancreatic cancer have not been studied yet.

**Fig. 9:**
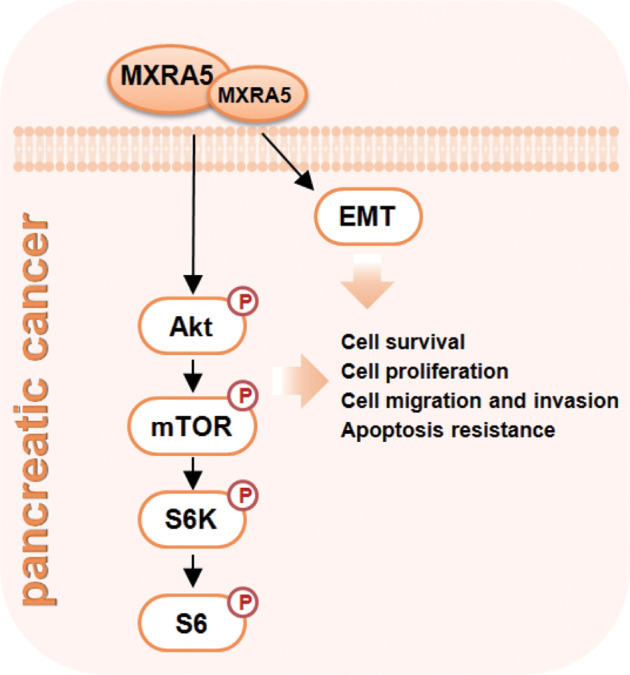
The proposed signaling carton of the present study.

Here we revealed that MXRA5 could be a novel and important oncogenic gene for pancreatic cancer. The bioinformatics studies revealed that *MXRA5* transcripts are significantly elevated in pancreatic cancer tissues, correlating with the poor overall survival, high T-stage, N1 and pathologic stage. *MXRA5* mRNA and protein expression is elevated in microarray pancreatic cancer tissues and various pancreatic cancer cells. In primary and established pancreatic cancer cells, MXRA5 depletion, using shRNA or CRISPR/Cas9 strategies, robustly suppressed cell viability, cell cycle progression, proliferation and motility, while provoking apoptosis. Contrarily, ectopic overexpression of MXRA5 accelerated growth and migration of pancreatic cancer cells. Moreover, overexpression of MXRA5 downregulated E-Cadherin, but upregulated N-Cadherin and Vimentin, indicating a role of MXRA5 in EMT progression (see Fig. 9). Bioinformatics analysis and protein chip analysis further supported a potential function of MXRA5 in EMT. In vivo, the growth of MXRA5 KO PANC-1 xenografts was largely inhibited in nude mice. Moreover, intratumoral injection of aav-packed MXRA5 shRNA potently inhibited primary pancreatic cancer cell growth in nude mice. Contrarily MXRA5 overexpression promoted primary pancreatic cancer cell growth in nude mice. Therefore, MXRA5 could be an important therapeutic oncotarget of pancreatic cancer (see Fig. 9).

Multiple signaling pathways are vital for pancreatic cancer initiation and progression, including RAF/MEK/ERK, PI3K/Akt/mTOR, Wnt/Notch and NFκB signaling etc [59–64]. Dysregulation of these pathways promotes cancer cell growth, progression, migration/invasion, and angiogenesis as well as apoptosis resistance [61]. A significant percentage of pancreatic cancers have increased PI3K/Akt cascade activation [60, 65, 66]. Treatment with Akt/mTOR inhibitors could potently arrest pancreatic cancer cell growth [67, 68].

Here bioinformatics studies and protein chip analyses of MXRA5-overexpressed pancreatic cancer cells revealed that MXRA5-associated DEGs and differentially DEPs are both enriched in PI3K-Akt-mTOR cascade. Indeed, Akt-mTOR activation was largely inhibited by MXRA5 shRNA or knockout in primary pancreatic cancer cells, but was augmented following ectopic overexpression of MXRA5. In addition, Akt-mTOR activation was also largely inhibited in the MXRA5-depleted pancreatic cancer xenografts. Thus, MXRA5-driven pancreatic cancer cell growth is possibly due to, at least in part, by promoting Akt-mTOR signaling (see Fig. 9).

## Conclusion

Identification of novel and key oncogenic molecules is extremely important for the diagnostic and prognostic determination for pancreatic cancer. We here propose that overexpressed MXRA5 could be an important protein for pancreatic cancer progression, representing as a promising therapeutic target.

## Supplementary information

Supplementary Figures

Author contribution form

aj-checklist form

## Data Availability

All data are available upon request.
