# Supplementary material for: Identification of matrix-remodeling associated 5 as a possible molecular oncotarget of pancreatic cancer
Source: Cell Death Dis. 2023 Feb 24;14(2):157. doi: 10.1038/s41419-023-05684-5 (PMC9958022; doi:10.1038/s41419-023-05684-5)
Supplement: Supplementary file 1 — Supplementary Figures [file 41419_2023_5684_MOESM1_ESM.pdf]

Figure S1.

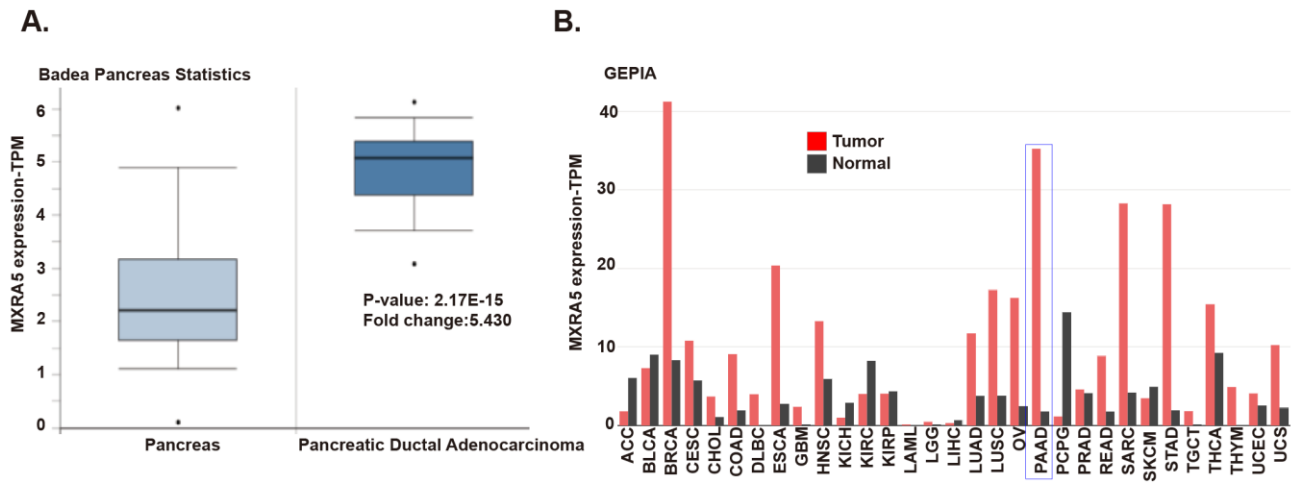

Figure S1. MXRA5 overexpression in pancreatic cancer tissues. *MXRA5* expression in pancreatic cancer tissues from the Oncomine database (www.oncomine.org) was retrieved (**A**). GEPIA online tool shows *MXRA5* expression in different cancer tissues ("Tumor") and corresponding normal tissues ("Normal") (**B**).

Figure S2

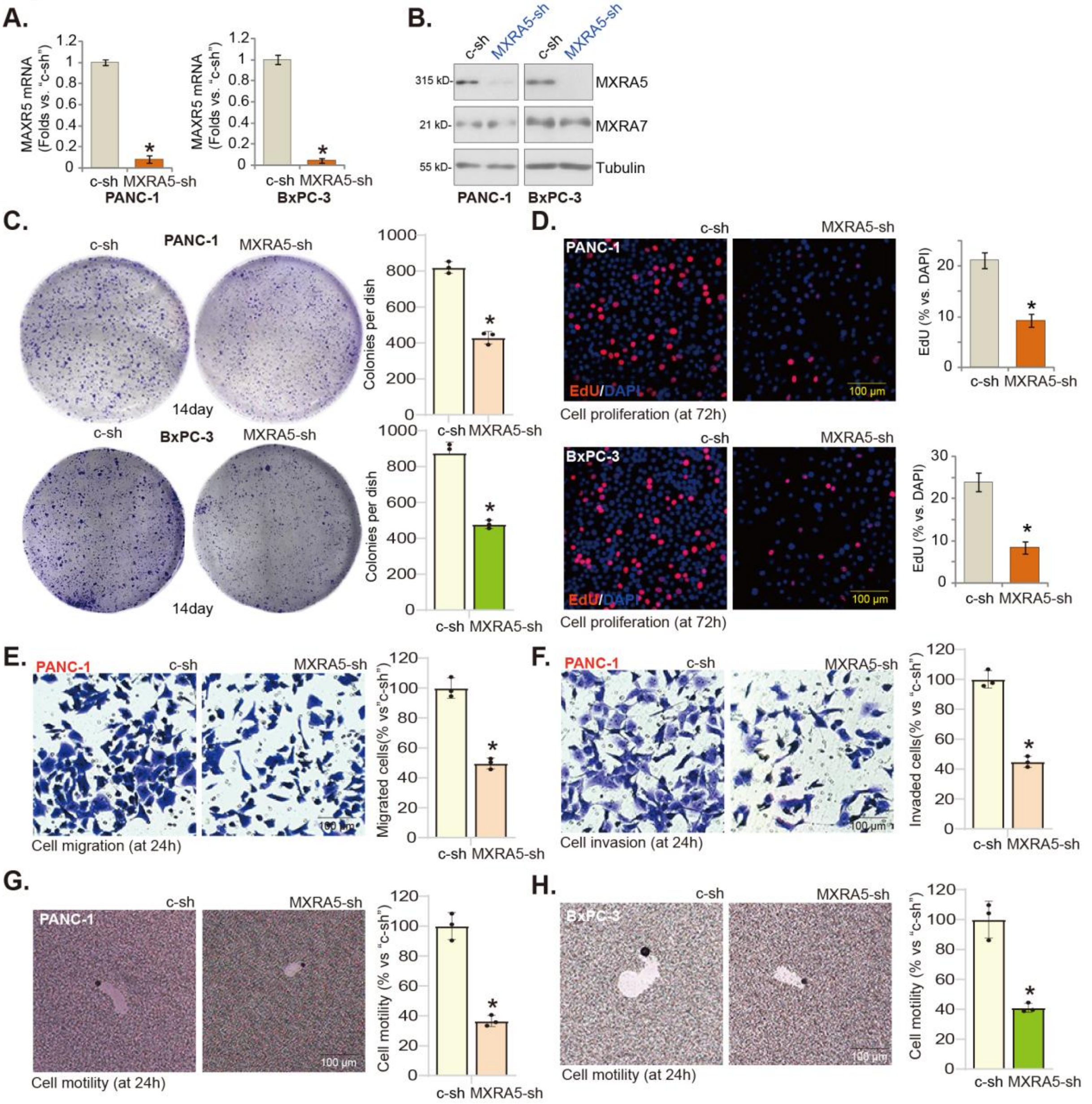

Figure S2. **MXRA5 downregulation exerts anti-tumorigenic activity in human pancreatic cancer cells.** Puromycin-selected pancreatic cancer cell lines (PANC-1 and BxPC-3), with MXRA5-sh-S1 ("MXRA5-sh") or the scramble control shRNA ("c-sh"), were cultured, and expression of listed genes and proteins was shown (**A** and **B**); Cells were further cultured for applied time periods, colony formation (**C**) and cell proliferation (**D**) as well as cell migration, invasion (**E** and **F**) and motility (**G** and **H**) were tested by the described assays, with results quantified. Data were presented as mean ± standard deviation (SD, n= 5). *$\boldsymbol{P}$ <0.05 vs. "c-sh" cells. All experiments were repeated for five times with similar results obtained. Scale bar = 100 μm.

Figure S3

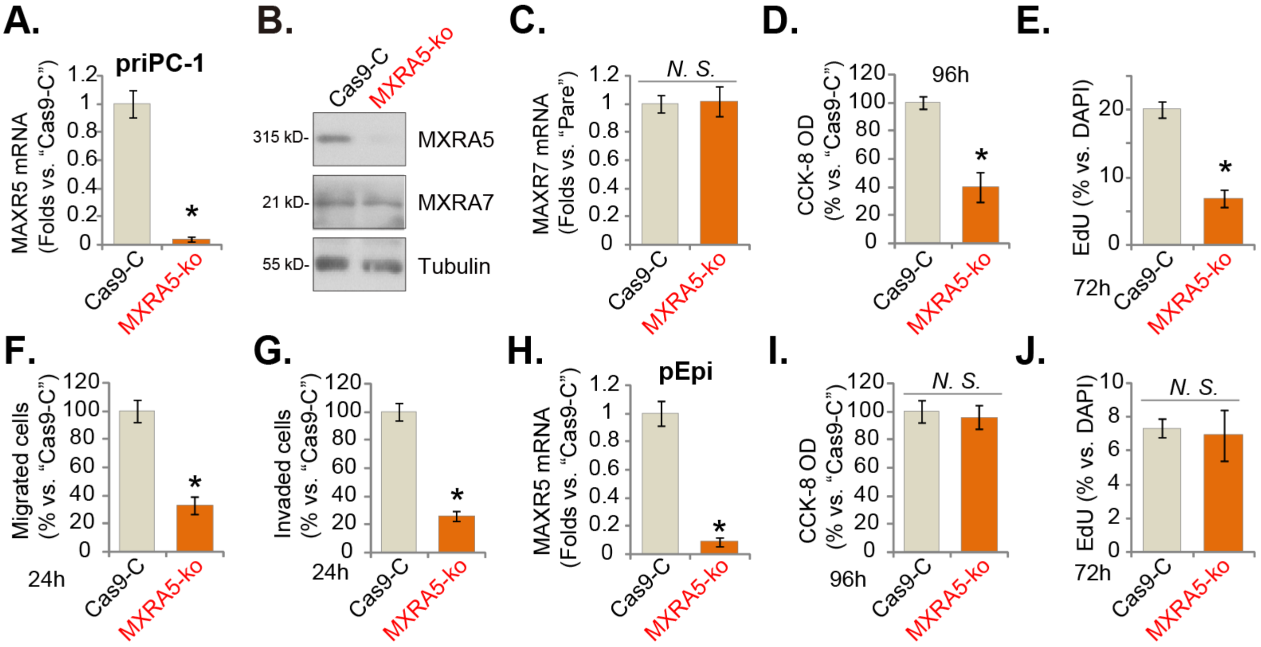

Figure S3. MXRA5 knockout potently inhibits pancreatic cancer cell progression *in vitro*. Puromycin-selected priPC-1 primary human pancreatic cancer cells (**A**-**G**), with sgRNA-CRISPR/dCas-9 MXRA5-KO lentiviral construct ("MXRA5-ko") or Cas9 control construct ("Cas9-C"), were established, and expression of listed genes and proteins was shown (**A**-**C**); Cells were further cultivated for indicated time periods, and cell viability (**D**), and EdU incorporation (**E**) as well as *in vitro* cell migration (**F**) and invasion (**G**) were tested using the described methods, and results were quantified. Puromycin-selected pEpi epithelial cells, expressing the sgRNA-CRISPR/dCas-9 MXRA5-KO lentiviral construct ("MXRA5-ko") or Cas9-C construct, were established. *MXRA5* mRNA expression was shown (**H**). Cells were further cultivated for indicated time periods, cell viability (**I**) and proliferation (**J**) were examined, and results were quantified. Error bars stand for mean ± standard deviation (SD, n=5). * P < 0.05 versus "Cas9-C" cells. "*N.S.*" stands for P > 0.05. Experiments in this figure were repeated five times.

Figure S4: The uncropped blotting images of the study

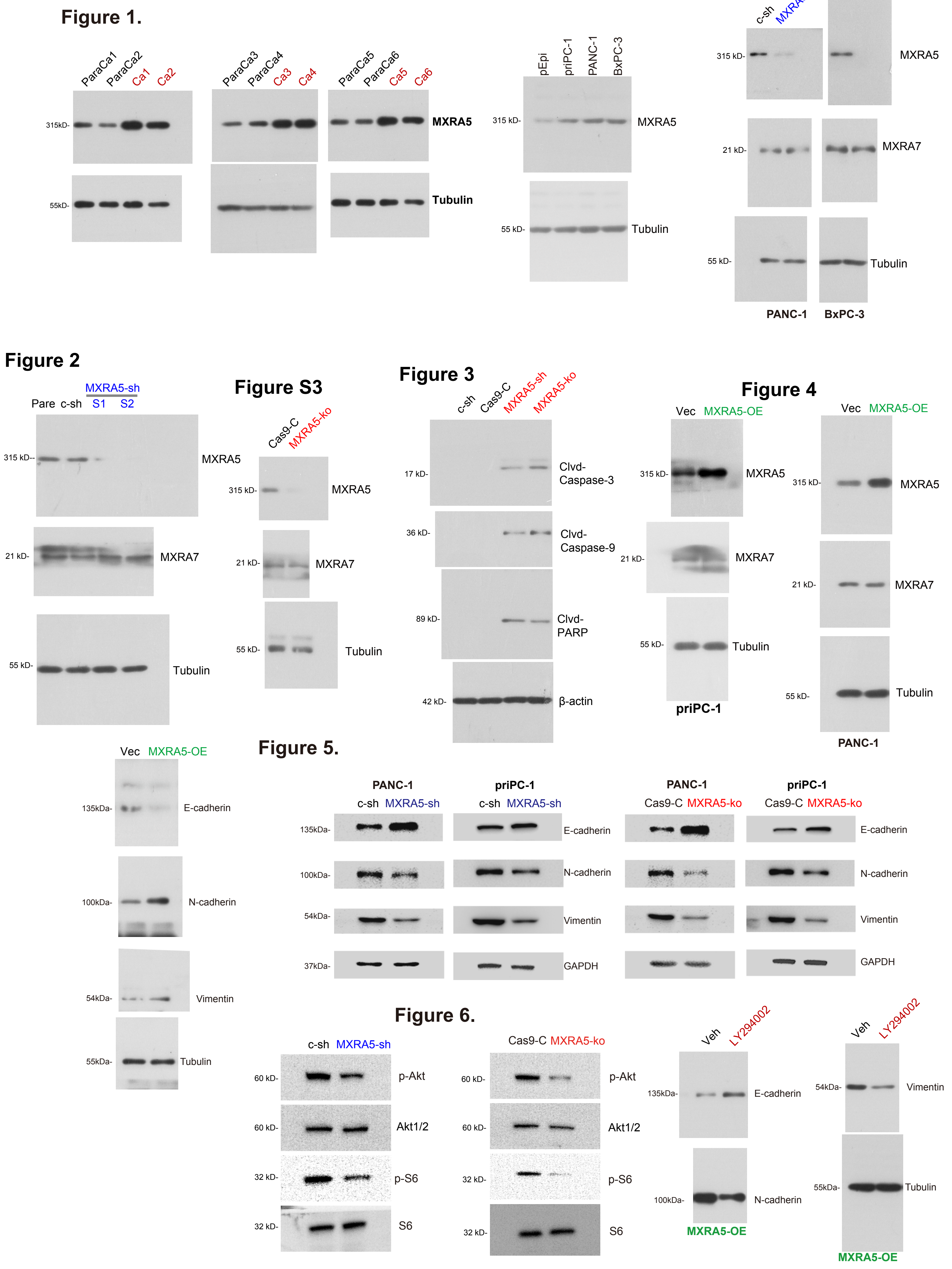

Figure S4-Continued

Figure S5.

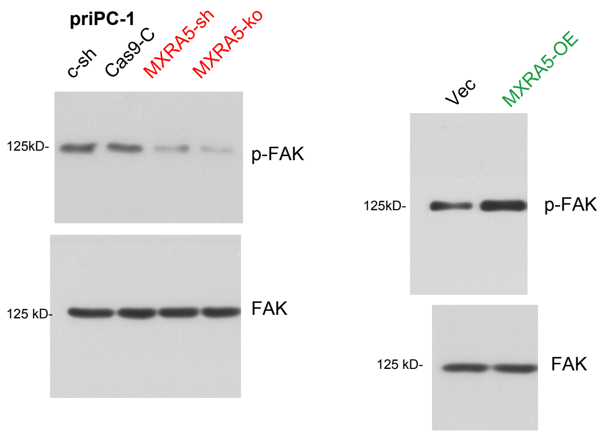

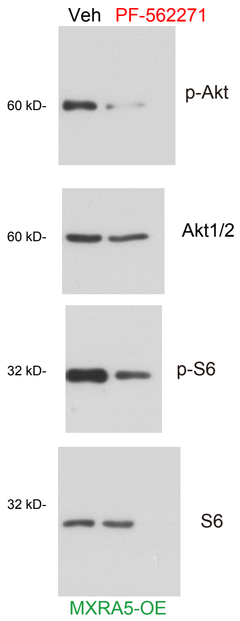

Figure 7.

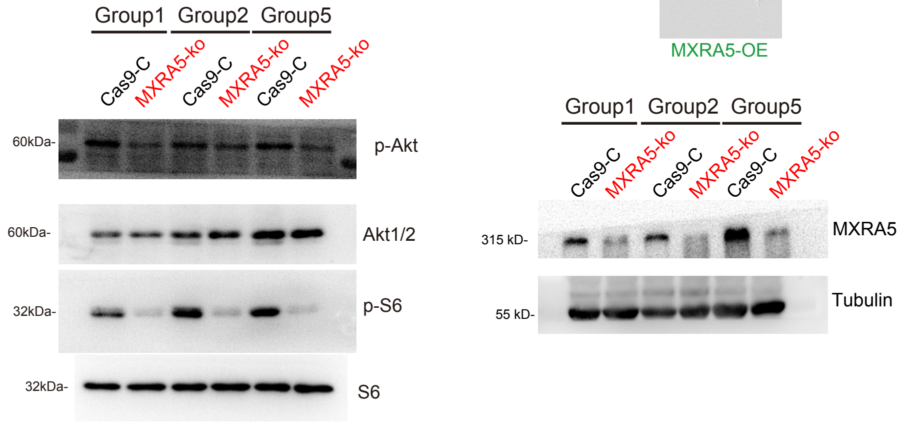

Figure 8

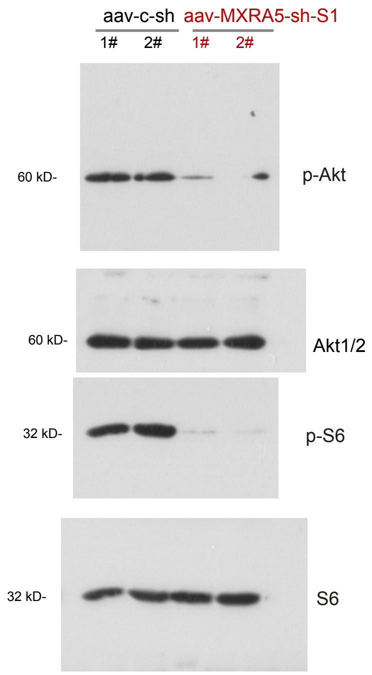

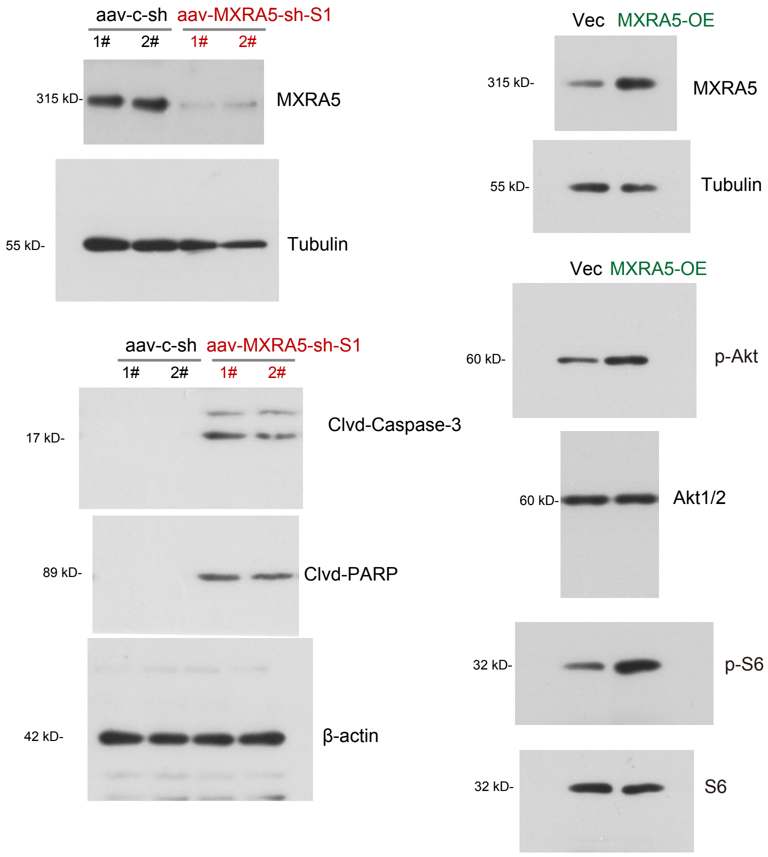

Figure S5.

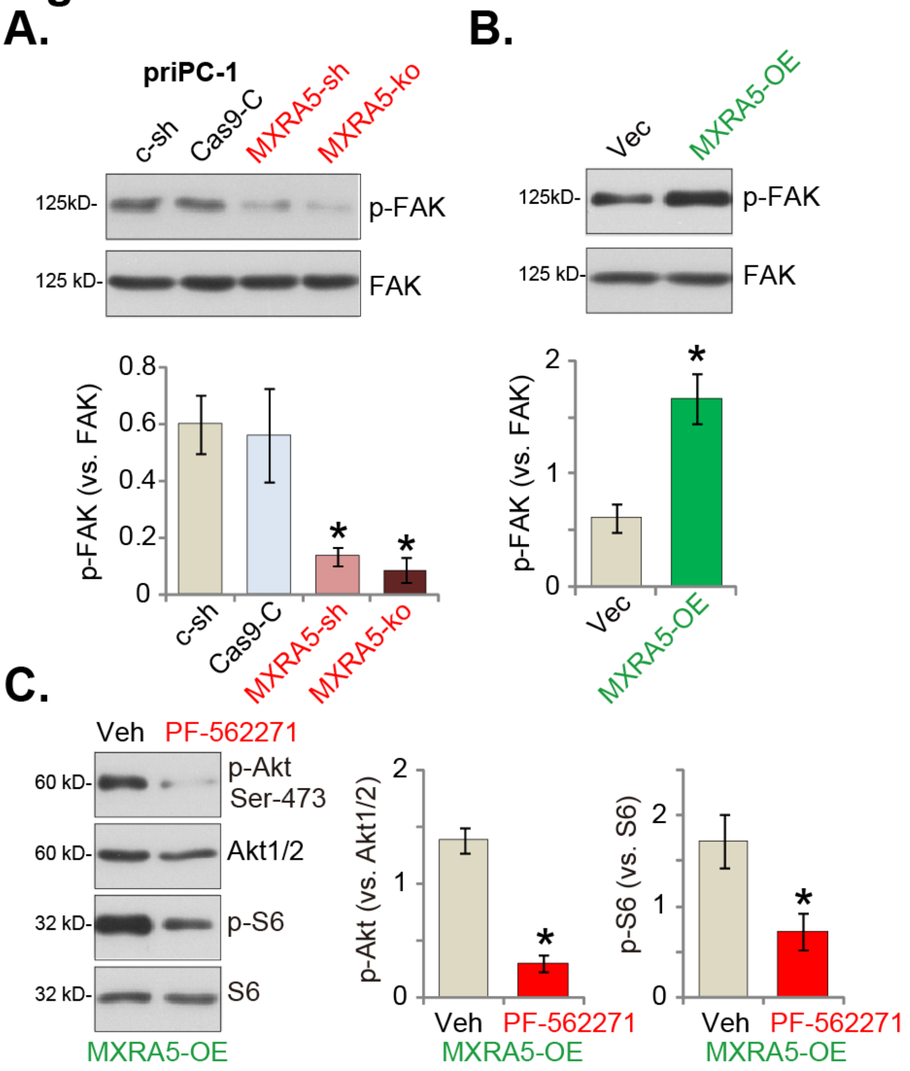

Figure S5. FAK is important for MXRA5-promoted Akt-mTOR activation in pancreatic cancer cells. Puromycin-selected priPC-1 cells with MXRA5-sh-S1 ("MXRA5-sh"), the scramble control shRNA ("c-sh"), the sgRNA-CRISPR/dCas-9 MXRA5-KO lentiviral construct ("MXRA5-ko"), Cas9 control construct ("Cas9-C"), the lentiviral MXRA5 overexpression construct ("MXRA5-OE") or the corresponding vector ("Vec") were cultured, and listed proteins tested (**A** and **B**). The OE-MXRA5 priPC-1 cells were treated with the FAK inhibitor PF-562271 (250 nM) or vehicle control (0.1% DMSO, "Veh") for 6h, expression of listed proteins was shown (**C**). Error bars stand for mean ± standard deviation (SD, n=5). * $P <$ 0.05 versus "Vec"/"c-sh" cells or "Veh" treatment. Experiments in this figure were repeated five times.
