# Supplementary material for: Identification of matrix-remodeling associated 5 as a possible molecular oncotarget of pancreatic cancer
Source: Cell Death Dis. 2023 Feb 24;14(2):157. doi: 10.1038/s41419-023-05684-5 (PMC9958022; doi:10.1038/s41419-023-05684-5)
Supplement: Supplementary file 2 — Author contribution form [file 41419_2023_5684_MOESM2_ESM.pdf]

DECLARATION OF CONTRIBUTIONS TO ARTICLE

ADMC

Manuscript Number: CDDIS-22-5315R

Journal Name: *Cell Death & Disease* (the 'Journal')

Proposed Title of the Contribution:

Identification of matrix-remodeling associated 5 as a possible molecular oncotarget of pancreatic cancer (the 'Contribution)

Author(s):

Shi-qing Peng, Xiao-ren Zhu, Ming-zhi Zhao, Yi-fan Zhang, An-ran Wang, Min-bin Chen, Zhen-yu Ye (the 'Authors')

For all *CDDis* articles, each person named as an author in the published version must be able to show he or she has contributed substantially to the article.

Authorship credit should be based on 1) substantial contributions to conception and design, acquisition of data, or analysis and interpretation of data; 2) drafting the article or revising it critically for important intellectual content; and 3) final approval of the version to be published. Authors should meet conditions 1, 2 and 3.

Any person who cannot be shown to have made a substantial contribution to the article cannot be listed as an author in the final version. The name of any person who is deemed to have made a minor contribution can, however, appear in the Acknowledgments section of the article.

Please complete the table below to indicate the contributions of all named authors to the manuscript.

Author Full Name:	Specification of Contribution to the Manuscript:
Shi-qing Peng	Functional assays in cancer and normal cells, establishing xenografts in mice, testing signalings in cells and tissues and performing cell death/apoptosis assays, drafting the article and revising it, with final approval of the version submitted to the journal.
Xiao-ren Zhu	Functional assays in cancer and normal cells, establishing xenografts in mice, testing signalings in cells and tissues and performing cell death/apoptosis assays, drafting the article and revising it, with final approval of the version submitted to the journal.
Ming-zhi Zhao	Functional assays in cancer and normal cells, establishing xenografts in mice, testing signalings in cells and tissues and performing cell death/apoptosis assays, drafting the article and revising it, with final approval of the version submitted to the journal.
Yi-fan Zhang	Functional assays in cancer and normal cells, establishing xenografts in mice, testing signalings in cells and tissues and performing cell death/apoptosis assays, drafting the article and revising it, with final approval of the version submitted to the journal.
An-ran Wang	Functional assays in cancer and normal cells, establishing xenografts in mice, testing signalings in cells and tissues and performing cell death/apoptosis assays, drafting the article and revising it, with final approval of the version submitted to the journal.
Min-bin Chen	Study conception and design, and data analysis, Figure organization, involved in drafting the article and revising it critically for important intellectual content, and with final approval of the version submitted to the journal.
Zhen-yu Ye	Study conception and design, and data analysis, Figure organization, involved in drafting the article and revising it critically for important intellectual content, and with final approval of the version submitted to the journal. .

Please complete the table below to indicate the contributions of all named authors to the figures.

Figure 1:

Shi-qing Peng, Xiao-ren Zhu, Ming-zhi Zhao, Yi-fan Zhang, An-ran Wang, Min-bin Chen and Zhen-yu Ye

Figure 2:

Shi-qing Peng, Xiao-ren Zhu, Ming-zhi Zhao, Yi-fan Zhang, An-ran Wang, Min-bin Chen, Zhen-yu Ye

Figure 3:

Shi-qing Peng, Xiao-ren Zhu, Ming-zhi Zhao, Yi-fan Zhang, An-ran Wang, Min-bin Chen, Zhen-yu Ye

Figure 4:

Shi-qing Peng, Xiao-ren Zhu, Ming-zhi Zhao, Yi-fan Zhang, An-ran Wang, Min-bin Chen, Zhen-yu Ye

Figure 5:

Shi-qing Peng, Xiao-ren Zhu, Ming-zhi Zhao, Yi-fan Zhang, An-ran Wang, Min-bin Chen, Zhen-yu Ye

Figure 6:

Shi-qing Peng, Xiao-ren Zhu, Ming-zhi Zhao, Yi-fan Zhang, An-ran Wang, Min-bin Chen, Zhen-yu Ye

Figure 7-9

Shi-qing Peng, Xiao-ren Zhu, Yi-fan Zhang, An-ran Wang, Min-bin Chen and Zhen-yu Ye

Signed for and on behalf of the Author(s):	Print Name:	Date:
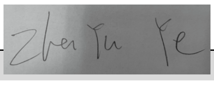	Zhen-yu Ye	01-18-2023
